# AI-Based Imaging Assessment of Body Composition in Oncology: A Step Toward Routine Clinical Practice Integration

**DOI:** 10.3390/healthcare14111476

**Published:** 2026-05-27

**Authors:** Elisa Mattavelli, Paolo Cotogni, Riccardo Caccialanza

**Affiliations:** 1 Clinical Nutrition and Dietetics Unit, Department of Oncology, Comprehensive Cancer Center, Fondazione IRCCS Policlinico San Matteo, 27100 Pavia, Italy; e.mattavelli@smatteo.pv.it (E.M.); r.caccialanza@smatteo.pv.it (R.C.); 2 Department of Internal Medicine and Medical Therapy, University of Pavia, 27100 Pavia, Italy; 3 Pain Management and Palliative Care, Department of Anesthesia, Intensive Care and Emergency, Molinette Hospital, University of Turin, 10126 Turin, Italy; 4 Department of Oncology and Hemato-Oncology, University of Milan, 20157 Milan, Italy

**Keywords:** body composition, artificial intelligence, oncology, computed tomography, nutritional assessment

## Abstract

**Highlights:**

**What are the main findings?**

- AI-based body composition analysis may enable rapid, automated and reproducible assessment from routine imaging, overcoming the limitations of manual approaches.
- Despite strong prognostic evidence, its routine integration into oncology clinical practice remains limited.

**What are the implications of the main findings?**

- AI-based body composition analysis may support early risk stratification, guiding tailored lifestyle intervention and anticancer treatment personalization.
- Bridging the implementation gap will require protocols standardization, robust validation and careful consideration of regulatory and ethical aspects.

**Abstract:**

In oncology, body composition (BC) provides clinically meaningful information beyond body mass index, capturing muscle and adipose tissue alterations associated with survival, treatment tolerance, surgical complications and quality of life. Although routine oncologic imaging is widely available, BC assessment remains poorly integrated into daily clinical practice, largely because conventional imaging-based approaches require time-consuming manual analyses, dedicated software and specialized expertise. Artificial intelligence (AI), particularly deep learning-based image segmentation, may automate BC analysis and generate rapid, reproducible, and scalable estimates from routinely acquired imaging, without increasing clinical workload. This opinion paper aims to examine AI-based BC analysis as a potential strategy to integrate BC into routine oncology workflows, outlining its potential clinical benefits and the aspects that need to be addressed before widespread implementation. AI-based BC analysis may improve nutritional assessment, refine clinical and nutritional risk stratification, and help identify patients at increased risk of treatment-related toxicity. In perspective, BC data may also support more personalized nutritional and physical activity interventions and contribute to muscle mass-informed anticancer treatment dosing strategies. Several gaps still limit its clinical implementation, including the need of robust external validation, standardized acquisition and analytical protocols, clinically meaningful cut-offs and ethical, and regulatory and data governance frameworks. AI-based BC analysis is therefore a promising but still evolving approach that may help translate BC from a prognostic marker into a clinically actionable tool in oncology.

## 1. Introduction

Body mass index (BMI) has long been employed as a simple and easily accessible parameter to assess nutritional status in oncology. However, its well-recognized limitations in distinguishing between fat mass and muscle mass restrict its clinical utility [1]. This limitation is particularly relevant, as among patients with cancer muscle depletion, adipose tissue alterations, sarcopenia, sarcopenic obesity, cancer-associated cachexia, and myosteatosis are frequent. These conditions may occur across a wide range of BMI values and may remain undetected by conventional anthropometric measures [2,3,4,5]. Relying solely on simple anthropometric measures to assess nutritional status in patients with cancer harbors the risk of failing to capture the full spectrum of at-risk patients. Indeed, Wang et al. recently reported a substantially higher prevalence of low muscle mass (25.4%) compared with a markedly lower prevalence of low BMI (4%) in a cohort of 126 patients with lung cancer, highlighting the limitations of BMI-based assessment in identifying patients with reduced muscle mass [6].

In this context, body composition (BC) has emerged as an accurate and clinically meaningful assessment, providing important phenotypic information with a highly relevant prognostic value.

A growing body of evidence has consistently demonstrated that BC parameters are strongly associated with key oncological outcomes, including overall survival, treatment-related toxicity [7], postoperative complications [8,9] and quality of life [10]. These findings supported the inclusion of BC assessment into oncology clinical guidelines and expert consensus statements [11,12,13,14]. Despite this strong prognostic evidence and its inclusion in oncology guidelines, BC assessment remains poorly integrated into routine oncology practice.

Cross-sectional imaging-based BC analysis is particularly relevant in oncology, as computed tomography (CT), magnetic resonance imaging (MRI) and positron emission tomography (PET) are already embedded in diagnostic, staging, response assessment and follow-up pathways. These routinely acquired images can be used opportunistically to quantify muscle and adipose tissue compartments without additional examination or patient burden.

However, its widespread adoption has been hindered by the complexity of the analysis workflow. In routine practice, imaging-based BC assessment typically requires retrieving cross-sectional imaging data, selecting the appropriate anatomical slice (most commonly at the third lumbar vertebra (L3) level), and performing manual segmentation, a process that is time-consuming and dependent on dedicated software and specialized expertise.

Artificial intelligence (AI), particularly deep learning-based image segmentation, may help address this implementation gap by enabling rapid, reproducible and scalable BC assessment from routinely acquired imaging. Recent reviews have mainly focused on the prognostic value of imaging-derived BC parameters or on the technical performance of AI-based segmentation models [15,16,17]. However, the clinical implementation gap remains less extensively addressed. Further clarification is needed on how AI-derived BC outputs should be interpreted, how they may define clinically meaningful patient phenotypes, how these metrics could be integrated into oncology workflows, and which methodological, clinical and organizational requirements are needed before they can support routine decision-making.

The aim of this opinion paper is to examine AI-based imaging BC analysis as a potential strategy to integrate BC assessment into routine oncology workflows. Moving beyond technical feasibility and prognostic associations and focusing on the clinical translation of AI-derived BC metrics by discussing how automated analysis may help bridge the gap between research and practice, which benefits it, may offer nutritional assessment, risk stratification, and tailored interventions, and which methodological, clinical, organizational, ethical and regulatory issues need to be addressed before widespread implementation.

## 2. Bridging the Evidence–Practice Gap Through Automated Imaging-Based Body Composition Analysis

The translation of imaging-based BC assessment into routine oncology practice has been limited by the operational complexity of conventional workflows. Although this approach has generated substantial prognostic evidence, it remains difficult to scale in high-volume clinical settings.

In this context, AI represents a promising development. Compared with traditional approaches, AI-based methods are paving the way for the automation of previously time-consuming and operator-dependent processes. Consequently, this approach harbors the advantage of substantially reducing the processing time, from approximately 10–15 min of expert manual work to near real-time computation, thereby optimizing resource utilization.

Most current approaches rely on AI models that first identify the appropriate anatomical slice, most commonly at the level of L3, and subsequently perform tissue segmentation. Although these steps are conceptually distinct, they operate within a coordinated pipeline, thus enabling fully automated analysis.

From a technical perspective, most automated approaches rely on deep learning. Convolutional neural network (CNNs) represents the broader family of models widely used for medical image segmentation for BC analysis [18]. U-Net and its variants are CNN-based fully convolutional architectures specifically designed for pixel- or voxel-level segmentation and are the most adopted models. More recently, transformer-based and hybrid CNN–transformer architectures have been explored to capture broader spatial relationships and contextual information with imaging data. However, model performance and generalizability depend not only on the architecture itself, but also on training data, annotation quality, imaging protocols and external validation.

To date, some validation studies have recently demonstrated high concordance between automated and manual imaging-based body composition analysis across different cancer types, reporting high Dice similarity coefficients (i.e., a statistic used to gauge the similarity or overlap between two samples or sets) for both the skeletal muscle (SM) and adipose tissue compartments. Importantly, these performances have been confirmed not only in internal validation settings but also in external cohorts, suggesting the potential robustness of these approaches [19,20,21,22,23]. Specifically, Delrieu et al., utilizing a U-Net architecture, reported a mean absolute error in the L3 selection of 4 mm in the internal cohort, and 5.5 mm in the external validation cohort was reported in a routinely performed CT scan from two cancer centers [20]. Dice similarity coefficients in the internal validation for BC compartments ranged from 0.86 to 0.94, with comparable coefficients in the external validation cohort [20]. Recent evidence from Potter et al. reported comparable findings in the oncological setting, employing a transformer-based segmentation model (Swin UNEt TRansformers) [19]. The reported concordance between manual and automated BC analysis suggests that this latter approach may be equally reliable in predicting cancer prognosis.

However, evidence linking AI-derived BC metrics to clinical outcomes remains less mature. Some studies have explored the prognostic value of automated BC parameters, but many were retrospective or based on relatively small cohorts. In a retrospective cohort of 45 patients with head and neck squamous cell carcinoma treated with immunotherapy, automated CT-based BC analysis showed that a higher skeletal muscle-to-bone ratio was independently associated with longer overall survival, while an early decrease in this ratio after three months was associated with worse survival [24]. Similarly, Matthias et al. investigated the impact of longitudinal postoperative changes in BC, assessed through automated techniques in 299 patients with gastroesophageal adenocarcinoma. Despite follow-up CT scans being available for only 55% of patients, they demonstrated that a reduction greater than 70% in visceral adipose tissue was associated with reduced survival [21].

In a large study, including 3345 patients with non-small cell lung cancer, automated BC-derived parameters were identified as significant prognostic factors [22].

More recently, Borys et al. evaluated a fully automated volumetric CT-derived sarcopenia index in a large pan-cancer cohort of 10,340 patients with solid tumors, with external validation in 439 patients. Higher sarcopenia index was associated with prolonged overall survival, and the index outperformed BMI and L3 skeletal muscle index in survival discrimination [25].

## 3. The Added Value of Body Composition Assessment in Oncology

The integration of AI-based BC analysis into routine oncology practice holds the potential to enhance both nutritional and clinical management of patients with cancer. Through the automated quantification of key metrics, including skeletal muscle (SM), visceral adipose tissue (VAT), abdominal subcutaneous adipose tissue (ASAT) and intermuscular adipose tissue (IMAT), this approach may enable the identification of clinically distinct body composition phenotypes with direct implications for patient management. In this framework, reduced SM may identify patients with low muscle mass, sarcopenia [2] or cachexia [4]; increased VAT and ASAT may reflect excess adiposity; the coexistence of reduced SM and excess adiposity may suggest the presence of sarcopenic obesity [3]; finally increased IMAT may provide information on myosteatosis, muscle fat infiltration and impaired muscle quality. This phenotypic approach is clinically relevant because patients with similar BMIs may have profoundly different muscle and fat distribution [5,6]. Accordingly, AI-based BC analysis may help identify patients who are not recognized as nutritionally vulnerable by BMI alone. The clinical value of BC phenotype identification lies in its potential to support individualized decision-making. In patients with low SM or myosteatosis, early referral of nutritional intervention, protein-enriched oral nutritional supplements (ONS) and physical activity programs may be prioritized. In patients with excess adiposity or sarcopenic obesity, interventions may need to combine nutritional optimization, preservation of lean mass, and tailored exercise rather than focusing only on weight reduction.

Several studies have explored the impact of these interventions among patients with cancer. A recent systematic review in patients with advanced colorectal cancer highlighted the potential role of protein-enriched ONS in preserving muscle mass, while evidence regarding physical activity remains heterogeneous, likely reflecting the challenges of implementing structured exercise programs in this population [26]. Similarly, a randomized controlled trial (RCT) in women with breast cancer undergoing neoadjuvant chemotherapy showed that the use of protein-enriched ONS resulted in increased lean mass and decreased fat mass, while BMI remained stable [27]. Another RCT in overweight/obese patients with prostate cancer showed how a lifestyle intervention combining nutrition and physical activity led to a significant reduction in fat mass while preserving muscle mass [28].

Body composition analysis extends beyond guiding lifestyle interventions, with potential implications for anticancer treatment dosing personalization. In patients with cancer, body composition compartments, especially muscle mass, have been associated with drug toxicity and treatment tolerance [7]. More recently, Liu et al. showed that AI-based quantification of SM and adipose tissue compartments improved prediction of dose-limiting toxicity in patients with colorectal cancer receiving chemotherapy [29]. Similarly, Besson et al. reported that AI-based BC predicted, with greater accuracy than body surface area, dose modification in patients undergoing neoadjuvant chemotherapy for rectal cancer [30]. These relations underscore the potential role of body composition in guiding dose adjustments of anticancer treatments, thus identifying patients who may benefit from adapted therapeutic strategies. In this context, BC could complement traditional clinical and laboratory parameters to refine patient selection and improve the balance between treatment efficacy and tolerability. Of note, the recent LEANOX trial demonstrated, in patients with cancer and low muscle mass, that oxaliplatin muscle mass-based dosing was associated with reduced toxicity compared to standard BSA dosing, without compromising treatment efficacy, as survival outcomes were comparable between groups [31]. Although AI-based body composition analysis may support future strategies for treatment personalization and dose optimization, these applications remain exploratory and require prospective validation before implementation in routine clinical practice.

## 4. From Innovation to Clinical Implementation

Several key challenges still need to be addressed to achieve a full implementation of AI-based BC analysis into routine oncology clinical practice (Table 1).

Considerable heterogeneity persists concerning the available AI-based models, each one harboring advantages and limitations. Pre-trained models offer scalability across institutions, but their performance may be affected by differences in imaging protocols and clinical context. Conversely, models trained on population-specific datasets may achieve higher accuracy within a given clinical environment, but at the same time, their performance may not translate beyond the original setting [20,21]. Hybrid strategies, combining pre-trained models with context-specific adaptation, may represent a promising compromise. Importantly, while these approaches reduce the need for large-scale manual annotation, they still require a limited amount of expert segmentation to adapt the model to the local data context [32].

Additionally, model reliability at the individual patient level is still a critical issue. Errors can occur in specific clinical scenarios, particularly in the presence of anatomical abnormalities or altered imaging conditions. Misclassification may arise from incorrect slice selection or structural variations, with reported error rates in the range of 2–5% in surgical oncology cohorts [23].

The implementation of AI-based BC analysis in routine oncology care requires more than algorithmic accuracy and standardized protocols. It depends on the ability to embed these tools into existing radiological and clinical workflows, while ensuring standardization, reproducibility, interpretability and appropriate governance. Importantly, AI-based BC tools can be integrated into Picture Archiving and Communication System (PACS) and Radiology Information System (RIS) environments through DICOM-compatible pipelines, allowing automatic analysis of routinely acquired CT or MRI images and visualization of segmentation outputs and quantitative metrics within the radiology workflow. Early studies have demonstrated the feasibility of PACS-integrated AI-based BC analysis from CT images, supporting its potential use in clinical routine [33]. However, successful implementation requires harmonized imaging protocols, local validation, quality-control procedures, regulatory compliance, data protection safeguards and multidisciplinary agreement on how AI-derived metrics should inform clinical decisions.

A further challenge concerns the definition of clinically meaningful thresholds.

Several CT-derived L3 skeletal muscle index cut-offs have been proposed in oncology, particularly to identify low muscle mass or sarcopenia, and these values are widely used for risk stratification. However, available thresholds differ according to sex, BMI, ethnicity, tumor type, disease stage and the clinical outcome used to derive them. Therefore, although they represent a useful framework, they should currently be interpreted as supportive rather than prescriptive tools for clinical decision-making.

For adipose tissue compartments, including VAT and ASAT, population-based reference data are increasingly becoming available, but oncology-specific and treatment-specific thresholds remain less well established. These metrics may help characterize visceral adiposity, excess adiposity and sarcopenic obesity phenotypes, particularly when interpreted together with skeletal muscle and muscle quality, but they should not yet be used as stand-alone decision thresholds.

In this context, AI-based BC analysis, allowing large-scale, automated extraction of quantitative metrics, might facilitate the generation of robust and data-driven reference thresholds [34].

A further methodological point concerns the distinction between cross-sectional phenotyping and longitudinal assessment. Although a single CT or MRI slice at L3 correlates with whole-body skeletal muscle and visceral adipose tissue, it may not reliably capture weight-loss-associated changes in skeletal muscle over time. Therefore, L3 single-slice analysis should primarily be considered a tool for baseline phenotyping and risk stratification, while longitudinal changes require cautious interpretation and integration with clinical, nutritional and functional measures [35].

To date, AI-based BC analysis in the oncology setting is still limited to small cohorts. Large-scale multicenter, real-world validation across different oncological populations are still lacking. Robust validation is essential for the effective implementation of routine AI-based BC.

Finally, ethical and regulatory aspects must be carefully addressed. The increasing use of AI in clinical decision-making raises important issues related to algorithm transparency and potential biases arising from training data [36,37]. Ensuring that these tools are interpretable, equitable and used under appropriate clinical supervision will be essential to guarantee a safe and effective implementation.

## 5. Conclusions

The integration of body composition assessment into supportive care pathways represents a crucial step toward more comprehensive oncology care. Early identification of patients at nutritional risk may facilitate timely referral to clinical nutrition services and support tailored interventions. More broadly, this approach aligns with the concept of “precision oncology” in which treatment decisions are informed not only by tumor biology, but also by patient-specific physiological characteristics. However, before AI-based body composition analysis can be routinely implemented in oncology care, further multicenter validation, standardized workflows, clinically meaningful thresholds, and prospective evidence of clinical utility are required.

## Figures and Tables

**Table 1:** Practical requirements for implementing AI-based body composition analysis in routine oncology care.

Requirement	Implementation Issue
Imaging variability	CT/MRI protocol, slice thickness and scanner-related variability may affect measurements.
Standardization	Consistent L3 selection and segmentation of SM, VAT, ASAT and IMAT are needed.
Local validation	AI models should be tested in the target population before clinical use.
Quality control	Visual overlays and expert review are needed to detect segmentation or slice-selection errors.
PACS/RIS integration	DICOM-compatible integration may allow automated analysis and reporting within routine radiology workflows.
Ethics and regulation	Transparency, privacy, bias assessment, accountability and clinical supervision are essential.
Clinical utility	Prospective studies should show that AI-based decisions improve relevant outcomes.

## Data Availability

Data sharing is not applicable. No new data were created or analyzed in this study.

## Abbreviations

The following abbreviations are used in this manuscript:

BMI	Body Mass Index
BC	Body Composition
SM	Skeletal Muscle
VAT	Visceral Adipose Tissue
ASAT	Abdominal Subcutaneous Adipose Tissue
IMAT	Intramuscular Adipose Tissue
AI	Artificial Intelligence
CT	Computed Tomography
L3	Third Lumbar vertebra
MRI	Magnetic Resonance Imaging
PET	Positron Emission Tomography
ONS	Oral Nutritional Supplements
RCT	Randomized Controlled Trial
AIOM	Italian Association of Medical Oncology
